# MicroRNA‐30a ameliorates hepatic fibrosis by inhibiting Beclin1‐mediated autophagy

**DOI:** 10.1111/jcmm.13278

**Published:** 2017-08-01

**Authors:** Jianliang Chen, Yue Yu, Shu Li, Yuting Liu, Shu Zhou, Shouji Cao, Jie Yin, Guoqiang Li

**Affiliations:** ^1^ Department of Liver Surgery The First Affiliated Hospital of Nanjing Medical University Nanjing Jiangsu Province China; ^2^ Key Laboratory of Living Donor Liver Transplantation of Ministry of Public Health Nanjing Jiangsu Province China; ^3^ Department of General Surgery People's Hospital Jingjiang Jiangsu Province China; ^4^ Department of Pediatric Surgery Huai'an First Hospital Affiliated to Nanjing Medical University Huai'an Jiangsu Province China; ^5^ Department of Respiratory Medicine Jinling Hospital Nanjing Jiangsu Province China

**Keywords:** microRNA‐30a, hepatic stellate cells, liver fibrosis, Beclin1, autophagy

## Abstract

We explored the role of microRNA‐30a (miR‐30a) and the mechanism involved in hepatic fibrosis. MiR‐30a overexpression was achieved by miR‐30a mimics transfection in hepatic stellate cells (HSCs) (HSC‐T6, LX‐2), and miR‐30a agomir (ago‐miR‐30a) treatment in mice. MiR‐30a levels were measured using TaqMan miRNA assay system, and the localization of miR‐30a was detected by fluorescence *in situ* hybridization (FISH). The interaction of miR‐30a and Beclin1 was confirmed by dual‐luciferase reporter assay. Autophagic flux was analysed using tandem mRFP‐GFP‐LC3 fluorescence microscopy, electron microscopy and Western blot of LC3‐II/I ratio. MiR‐30a was notably down‐regulated in activated HSCs and LX‐2‐exosomes induced by TGF‐β1; overexpression of miR‐30a down‐regulated extracellular matrix (ECM), such as α‐SMA, TIMP‐1, and Collagen I expression, and suppressed cell viability in HSCs. MiR‐30a was significantly down‐regulated in hepatic fibrosis mice and overexpression of miR‐30a prevented BDL‐induced fibrogenesis, concomitant with the down‐regulation of ECM. MiR‐30a inhibited HSCs autophagy and increased lipid accumulation in HSCs and in mice fibrotic hepatic tissues. MiR‐30a inhibited its downstream effector of Beclin1 by direct targeting its 3′‐UTR region. Moreover, Knock‐down of Beclin1 by small interfering RNA (siRNA) inhibited HSC autophagy and activation in LX‐2 cells. In conclusion, miR‐30a is down‐regulated in hepatic fibrosis models and its overexpression prevents liver fibrogenesis by directly suppressing Beclin1‐mediated autophagy; therefore, miR‐30a may be a new potential therapeutic target for controlling hepatic fibrosis.

## Introduction

Liver fibrosis is an excessive scarring response to chronic liver damage characterized by the accumulation of extracellular matrix (ECM) proteins, mainly type I fibrillar collagen (Collagen I) 1. As a reversible disease, the liver fibrotic process can be attenuated or reversed by effective therapies, for example, weight loss and physical training for non‐alcoholic steatohepatitis (NASH), or antiviral treatment for chronic hepatitis B and C 2. Hepatic stellate cells (HSCs), formerly considered as lipocytes, play a pivotal role in the process of hepatic fibrosis, regardless of the potential aetiology 3. Once activated, HSCs develop into fibrogenic myofibroblast‐like cells (activated HSCs), which secrete α‐SMA (a hallmark for activated HSCs), TIMP‐1(a key molecule involved in the inhibition of HSC apoptosis) and Collagen I 3, 4, 5.

Among the characteristic features of stellate cell activation are the loss of HSC lipid droplet (LD) retinoid stores 6. Autophagy is required for LD breakdown and the loss of LDs by autophagy drives the fibrogenic response in HSCs 7. Selective reduction of autophagic activity increases LDs accumulation in HSCs and impairs HSC activation, thereby suppressing liver fibrogenic activity 8.

MicroRNAs (miRNAs) are non‐coding, short (22–61 nucleotides) RNA molecules that were first identified by Ambros *et al*. in 1993 9. MiRNAs induce target mRNA degradation and repress mRNA translation at the 3′‐untranslated region (3′‐UTR) 10. MiR‐30a, a member of the miR‐30 family, is located on human chromosome 6q.13 11, and is involved in most cellular processes, including mRNA transcription and protein translation 12. Du *et al*. 13 identified 18 down‐regulated and 19 up‐regulated miRNAs in fibrotic livers, of which miR‐30a was down‐regulated in a NASH model in C57BL/6 mice, and studies have reported that miR‐30a is a key regulator of myocardial fibrosis 14 and peritoneal fibrosis 15. Peng *et al*. 16 reported that miR‐30a can inhibit the epithelial–mesenchymal transition. Zhu *et al*. 17 reported that Beclin1, a key autophagy‐related gene, was a possible target for miR‐30a, which negatively regulated Beclin1 to inhibit autophagic activity. However, the relationships between miR‐30a, autophagy and hepatic fibrosis are still yet to understood.

In this study, we firstly examined the level of miR‐30a in the experimental hepatic fibrosis models *in vitro* and *in vivo*, which showed that miR‐30a was down‐regulated in activated HSCs and exosomes secreted from cultured LX‐2 cells (LX‐2‐exosomes) induced by TGF‐β1, and in fibrotic liver tissues. The functional role and therapeutic potential of miR‐30a in liver fibrogenesis were characterized *in vitro* and *in vivo* by miR‐30a mimics transfection into HSCs and miR‐30a agomir transfection into mice, respectively. Our results showed that miR‐30a overexpression directly down‐regulated Beclin1 expression and attenuated HSC activation. Moreover, LC3‐II/I ratio showed that miR‐30a overexpression inhibited autophagy. Particularly, we revealed that silencing of Beclin1 inhibited HSCs autophagy and activation in LX‐2 cells. In conclusion, overexpression of miR‐30a may ameliorate liver fibrosis by suppressing Beclin1‐mediated autophagy.

## Materials and methods

### Mouse liver fibrosis induced by BDL or CCL_4_

Male C57BL/6 mice, 6–8‐week age, were purchased from Shanghai Jiesijie Experimental Animal Co. Ltd (Pujiang, Shanghai, China) (SCXK2013‐0006). All mice were maintained under standard conditions at the animal house of Nanjing Medical University. Water and food were provided *ad libitum*. Experimental hepatic fibrosis was induced by BDL (for 2 weeks) 18 or CCl_4_ (10% in olive oil, 2 ml/kg, twice a week for 6 weeks) 19 application as previously described. All animal studies were approved by the Institutional Animal Care and Use Committee of Nanjing Medical University (IACUC protocol number: NJMU08‐092). All mice were executed by air embolism death method after the experiment.

### HSCs activated by TGF‐β1

TGF‐β1 is a characterized cytokine known to initiate HSC activation 3, and the activation of HSCs is a significant event in liver fibrosis. Immortalized HSCs (HSC‐T6, LX‐2) (FuDan IBS Cell Center, Shanghai, China) were cultivated in Dulbecco's modified Eagle medium (DMEM) (Sigma‐Aldrich, St. Louis, MO, USA) containing 10% foetal bovine serum (FBS) (Gibco; Thermo Fisher Scientific, Waltham, MA, USA), and treated with TGF‐β1(10 ng/ml) for 0, 12, 24 and 48 hrs, respectively for activation.

### MiR‐30a and si‐Beclin1 transfection *in vitro* or *in vivo*

The miR‐30a mimics and negative control (miR‐NC) were transfected into HSCs (HSC‐T6, LX‐2), the Beclin1 siRNA (si‐Beclin1) and negative control (si‐NC) were transfected into LX‐2 cells, using Lipofectamine 2000 (Invitrogen, Carlsbad, CA, USA). The miR‐30a agomir and agomir control (AC) were transfected into C57BL/6 mice with siRNA‐Mate™ transfection reagent (Gema, Shanghai, China) four times (twice a week for 2 weeks) 20. The consequences were shown in Table S1.

### Fluorescence *in situ* hybridization (FISH)

FISH‐based miR‐30a imaging was performed, based on a previously described protocol 21, and the 5′ biotin‐labelled probe (CTTCCAGTCGAGGATGTTTACA) against homo sapiens miR‐30a (hsa‐miR‐30a) was purchased from Gema.

### Dual‐luciferase reporter assay

Potential target of miR‐30a and Beclin1 was predicted by TargetScan (www.targetscan.org), PicTar (pictar.mdc‐berlin.de) and miRanda (www.microRNA.org). At 24‐hrs post‐transfection, luciferase activities were analysed in LX‐2 cells by the dual‐luciferase reporter assay 22 system (Promega, Madison, WI, USA).

### Masson's trichrome staining

Masson's trichrome staining was performed to evaluate liver fibrosis. Collagen deposition was quantitatively analysed for collagen volume fraction (CVF) in each slice using the following formula: CVF = collagen area/total area × 100%.

### Exosome isolation and identification

LX‐2 cells were cultured in DMEM containing 10% exosome‐depleted FBS. LX‐2‐exosomes were extracted using Ribo™ Exosome Isolation Reagent (for cell culture media) (RiboBio, Guangzhou, China), according to the manufacturer's recommendations. To test the exosomes, exosomal markers, CD63 and CD81 23 were analysed with monoclonal antibodies (BD Biosciences, Franklin Lakes, NJ, USA) by Accuri C6 Flow cytometry (FCM) (Becton, Dickinson and Company, New Jersey, USA). The size of exosomes was tested with ZETASIZER Nano series‐Nano‐ZS (Malvern, England).

### Analysis of autophagic flux

To analyse autophagic flux, LX‐2 cells were transfected with a tandem fluorescent mRFP‐GFP‐tagged LC3 plasmid 24 using Lipofectamine 2000. The expression of GFP and mRFP was visualized with Olympus FV1000 laser scanning confocal microscope (Olympus, Tokyo, Japan). Images were acquired using FV10‐ASW3.0 software. Yellow (merge of GFP signal and RFP signal) puncta represented early autophagosomes, while red (RFP signal alone) puncta indicate late autolysosomes. Autophagic flux was evaluated by the colour change of GFP/mRFP.

### Real‐time PCR (RT‐PCR) analysis

The levels of U6 and miR‐30a were tested using the TaqMan miRNA assay system (Life Technologies Corporation, Shanghai, China). To detect mRNA expression, complementary DNA was synthesized using RT‐Master Mix (TaKaRa‐Bio, Shiga, Japan) and reverse transcription was followed by RT‐PCR using the StepOnePlus RT‐PCR system (Applied Biosystems, Foster City, CA, USA). Primers used in this study are shown in Table S2.

### Western blot analysis and immunohistochemistry

For Western blot analysis and immunohistochemistry, the specific antibodies were used as follows: α‐SMA (Cell Signaling Technology, Danvers, MA, USA), TIMP‐1 (Santa Cruz, CA, USA), Collagen I (Southern Biotech, Birmingham, AL, USA), Beclin1 (Cell Signaling Technology) and LC3 (Cell Signaling Technology).

### Statistical analysis

The consequences were expressed as the mean ± S.E.M. Analyses were performed with GraphPad Prism 6.0 (GraphPad Software Inc, La Jolla, CA, USA). *P *<* *0.05 was considered significant.

## Results

### MiR‐30a is down‐regulated in activated HSCs and LX‐2‐exosomes induced by TGF‐β1

**Figure 1:**
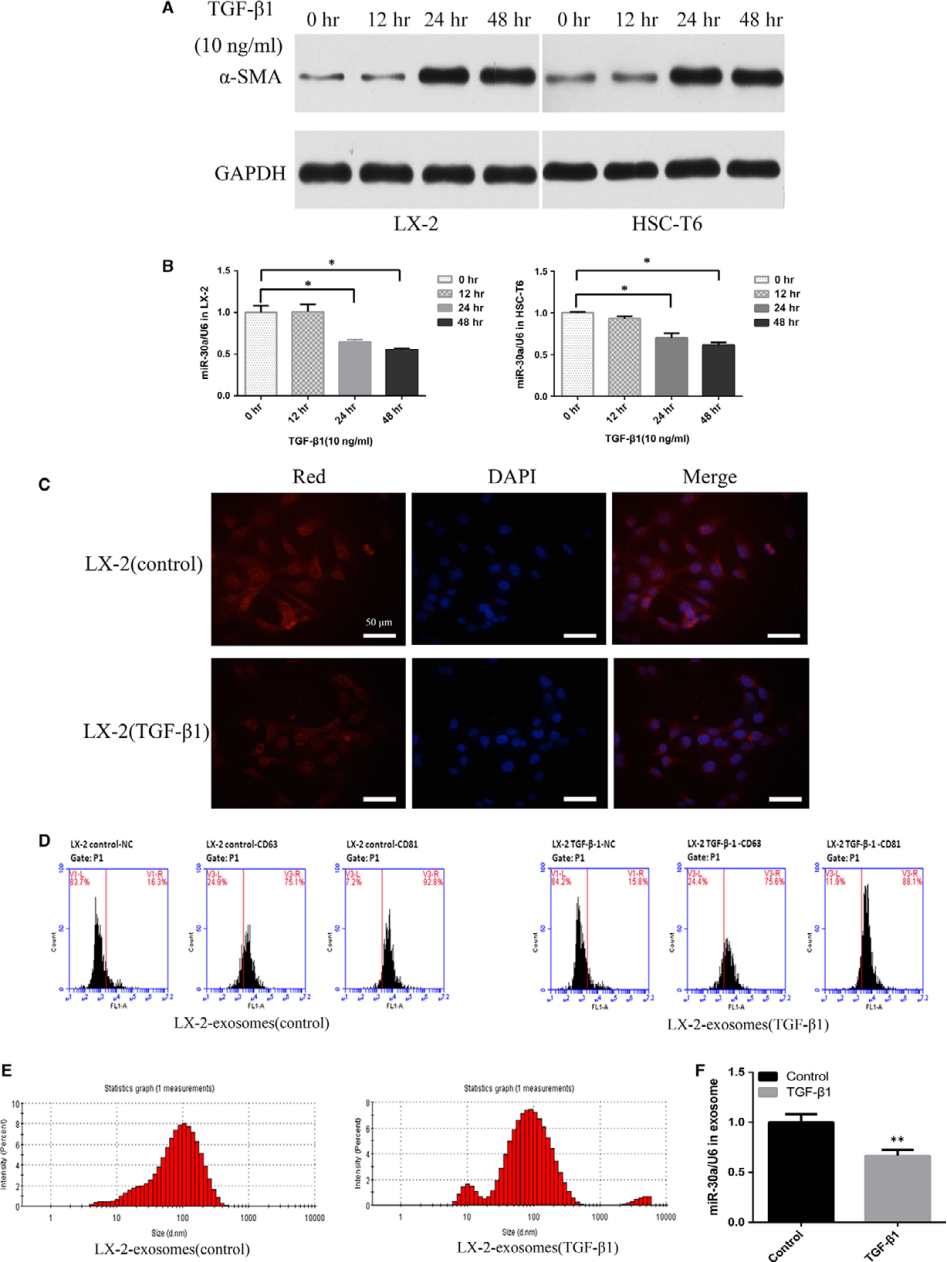
MiR‐30a is down‐regulated in activated HSCs and TGF‐β1‐treated LX‐2‐exosomes. HSCs were treated with TGF‐β1. (**A**) α‐SMA levels were detected by Western blotting. (**B**) miR‐30a levels were examined by TaqMan miRNA assay (*P < 0.05). (**C**) Detection of miR‐30a in LX‐2 cells using FISH, miR‐30a (Red) and nucleus (blue). (**D**) FCM analysis of surface markers (CD63 and CD81) on LX‐2‐exosomes. (**E**) Size detection of LX‐2‐exosomes. (**F**) MiR‐30a levels in LX‐2‐exosomes were examined by TaqMan miRNA assay. (**P < 0.01 *versus* the control group).

As shown in Figure 1A, TGF‐β1‐induced HSCs activation in both LX‐2 and HSC‐T6 cells was associated with α‐SMA up‐regulation. However, miR‐30a level showed a time‐dependent decrease in response to TGF‐β1 in HSCs (Fig. 1B). FISH data showed that miR‐30a expression mainly decreased in the cytoplasm of TGF‐β1‐treated LX‐2 cells, compared with the control group (treated with culture medium only) (Fig. 1C). The miR‐30a expression in LX‐2‐exosomes was further investigated, and FCM analysis (Fig. 1D) and size detection (Fig. 1E) showed that LX‐2‐exosomes expressed exosomal markers (CD63 and CD81) 23 and had diameters ranging from 20 to 200 nm. MiR‐30a expression was down‐regulated in LX‐2‐exosomes treated with TGF‐β1(by 0.66 ± 0.06‐fold *versus* the control group, *P *<* *0.01) (Fig. 1F). In conclusion, our results showed that miR‐30a was down‐regulated in HSCs and LX‐2‐exosomes induced by TGF‐β1.

### Overexpression of miR‐30a reverses the activation of HSCs and inhibits HSCs proliferation *in vitro*

**Figure 2:**
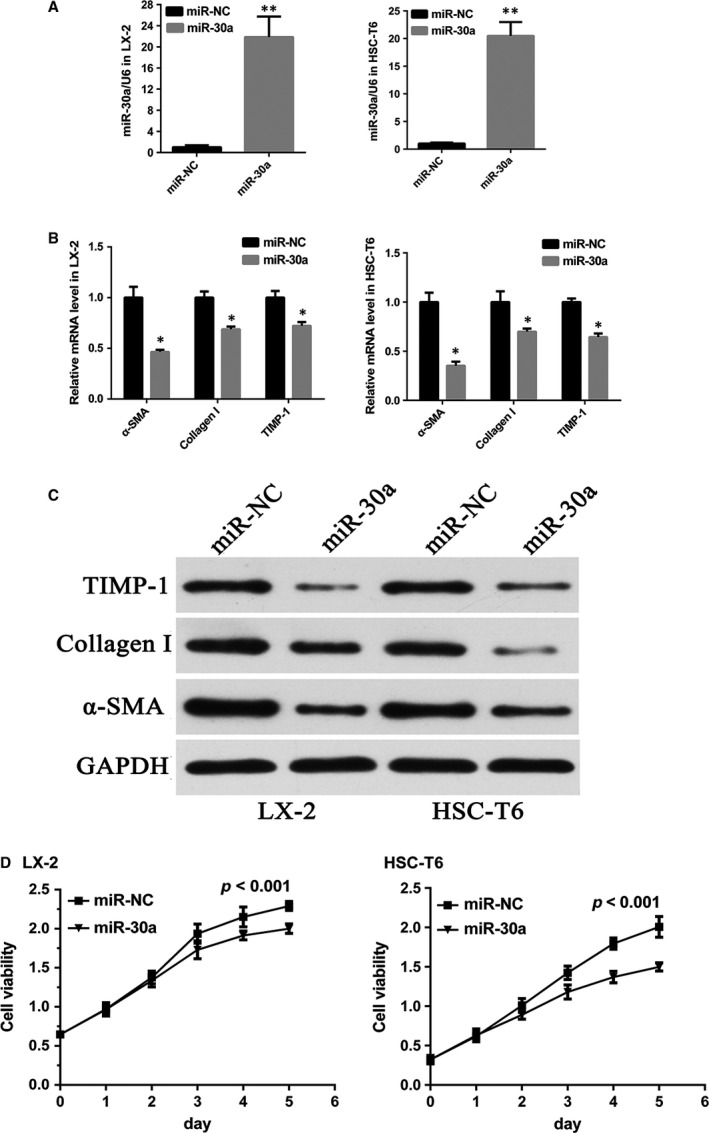
MiR‐30a reverses the activation of HSCs. (**A**) Overexpression of miR‐30a in HSCs. (**P < 0.01) α‐SMA, TIMP‐1 and Collagen I levels in miR‐30a overexpressing HSCs were tested by (**B**) RT‐PCR (*P < 0.05) and (**C**) Western blot. (**D**) MiR‐30a inhibited HSC cell growth.

We examined whether miR‐30a modulated the activation of HSC *in vitro*. The miR‐30a levels were significantly higher in LX‐2 (by 21.8 ± 3.9‐fold *versus* the control group, *P *<* *0.01) and HSC‐T6 (by 20.4 ± 2.5‐fold *versus* the control group, *P *<* *0.01) after transfected with miR‐30a mimics (Fig. 2A). MiR‐30a overexpression resulted in a pronounced down‐regulation of molecular factors involved in HSC activation, including α‐SMA, TIMP‐1, and Collagen I at both the mRNA (Fig. 2B) and protein (Fig. 2C) levels, suggesting the suppressed activation of HSCs by miR‐30a. Additionally, as determined by cell viability assay, miR‐30a also notably suppressed cell growth in both LX‐2 and HSC‐T6 cells (Fig. 2D).

### Overexpression of miR‐30a inhibits autophagy in HSCs by directly suppressing Beclin1 expression

**Figure 3:**
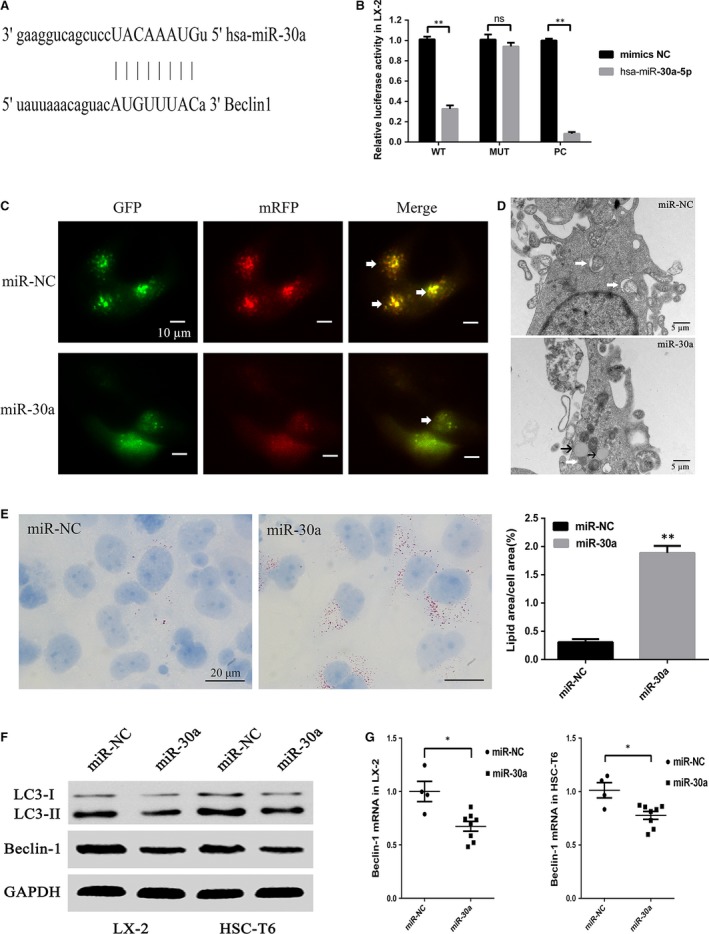
MiR‐30a inhibits HSC autophagy. (**A**) Putative seed‐matching sites between miR‐30a and Beclin1. (**B**) Luciferase reporter assay was performed on LX‐2 cells to detect the relative luciferase activities of WT and MUT Beclin1 reporters. (**P < 0.01) (**C**) Autophagic flux in LX‐2 cells. Arrows indicate autophagosomes. (scale bar = 10 μm) (**D**) Electron micrographs in LX‐2 cells transfected with miR‐30a, white arrows indicate autophagic vacuoles, black arrows indicate lipid droplets. (scale bar = 5 μm) (**E**) Lipid content analysis in LX‐2 cells treated with miR‐30a (scale bar = 20 μm) (**P < 0.01) (**F**) MiR‐30a down‐regulated Beclin1 and LC3‐II/I in HSCs. (**G**) Beclin1 levels in miR‐30a overexpressing HSCs. (*P < 0.05).

We found that the 3′‐UTR of Beclin1 contains putative binding sites for miR‐30a (Fig. 3A). The results showed that, miR‐30a repressed the reporter activity of the transcript containing wild‐type 3′‐UTR of Beclin1 (by 0.32 ± 0.02‐fold *versus* miR‐NC, *P *<* *0.01), indicating the direct regulation of miR‐30a on Beclin1 (Fig. 3B). RT‐PCR (Fig. 3G) and Western blot (Fig. 3F) showed that Beclin1 was down‐regulated in LX‐2 (by 0.67 ± 0.56‐fold *versus* the control group, *P *<* *0.05) and HSC‐T6 (by 0.77 ± 0.04‐fold *versus* the control group, *P *<* *0.05) cells transfected with miR‐30a mimics. The miR‐30a mimics decreased the number of autophagosomes in LX‐2 cells (Fig. 3C), suggesting that miR‐30a inhibited autophagy. There was a decrease in the number of autophagic vacuoles in LX‐2 cells treated with miR‐30a mimics (Fig. 3D). Moreover, Oil Red O (ORO) staining (by 5.56 ± 0.21‐fold *versus* the control group, *P *<* *0.01) (Fig. 3E) and electron microscopy (Fig. 3D) revealed an increased number of LDs in LX‐2 cells treated with miR‐30a mimics. MiR‐30a overexpression inhibited autophagy in HSC‐T6 and LX‐2 cells evidenced by decreased LC3‐II/I ratio (Fig. 3F). These results confirmed that Beclin1 is the target of miR‐30a and indicated that inhibition of Beclin1 expression by miR‐30a leads to the suppression of autophagic activity *in vitro*.

### Knock‐down of Beclin1 ameliorates HSCs autophagy and activation *in vitro*

**Figure 4:**
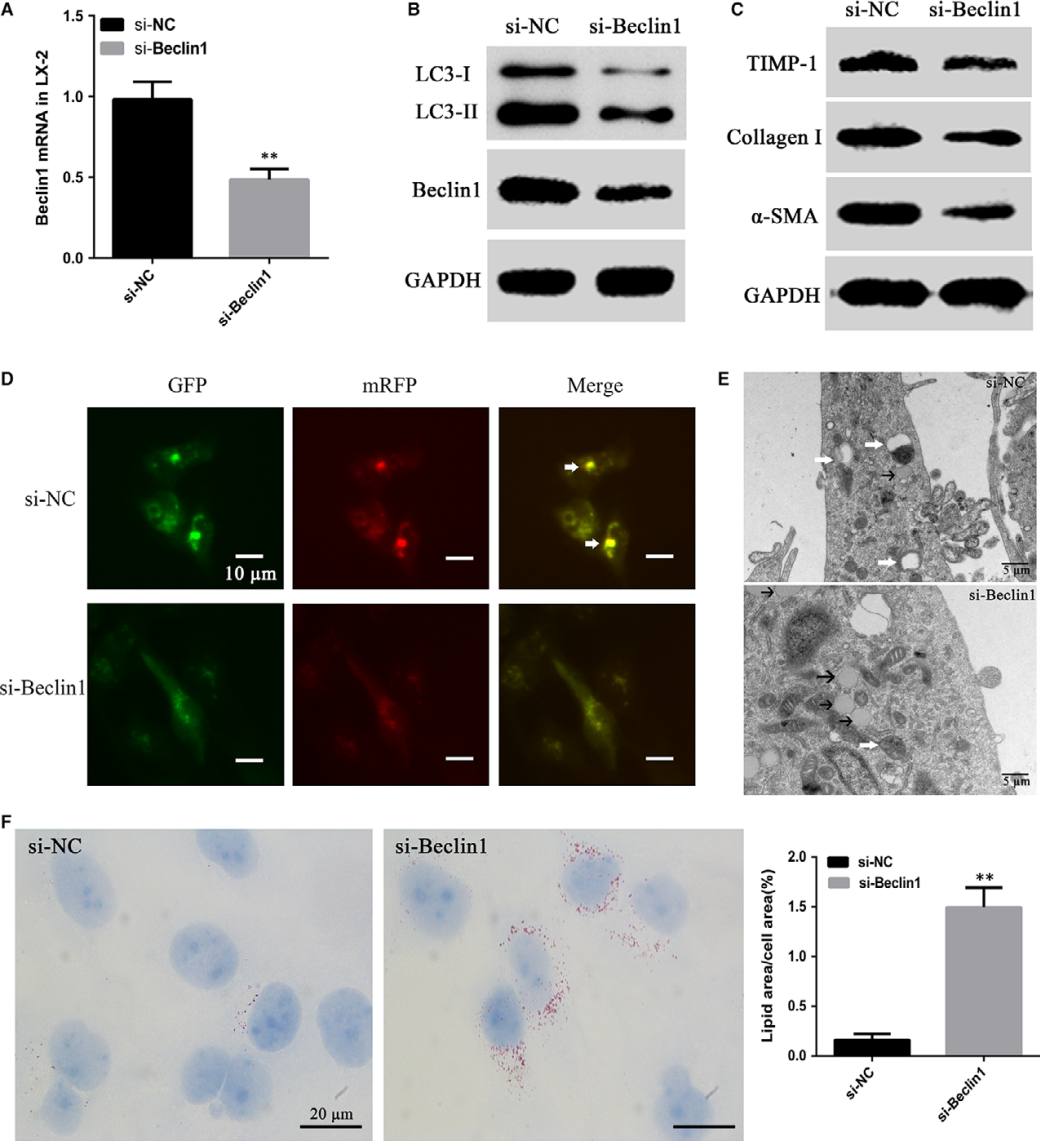
Knock‐down of Beclin1 suppresses the activation and autophagy of HSCs. (**A**) Beclin1 knock‐down efficiency in the LX‐2 cells was assessed by RT‐PCR and (**B**) Western blotting. (**P < 0.01) (**B** and **C**) si‐Beclin1 down‐regulates α‐SMA, TIMP‐1, Collagen I and LC3‐II/I in LX‐2 cells. (**D**) Autophagic flux in LX‐2 cells. Arrows indicate autophagosomes. (scale bar = 10 μm) (**E**) Electron micrographs in LX‐2 cells transfected with si‐Beclin1, white arrows indicate autophagic vacuoles, black arrows indicate lipid droplets. (scale bar = 5 μm) (**F**) Lipid content analysis in LX‐2 cells treated with si‐Beclin1 (scale bar = 20 μm) (**P < 0.01).

Knock‐down efficiency of Beclin1 was examined by RT‐PCR (by 0.47 ± 0.04‐fold *versus* the control group, *P *<* *0.01) (Fig. 4A) and Western blot (Fig. 4B). Knock‐down of Beclin1 strikingly down‐regulated α‐SMA, TIMP‐1 and Collagen I expression (Fig. 4C), indicating that knock‐down of Beclin1 inhibited the activation of HSCs. Knock‐down of Beclin1 in LX‐2 cells also markedly down‐regulated LC3‐II/I ratio (Fig. 4B) and decreased the amount of autophagosomes (Fig. 4D). There was also a decrease in the number of autophagic vacuoles in LX‐2 cells treated with si‐Beclin1 (Fig. 4E). ORO staining (by 9.33 ± 0.72‐fold *versus* the control group, *P *<* *0.01) (Fig. 4F) and electron microscopy (Fig. 4E) revealed an increased number of LDs in Beclin1 knock‐down LX‐2 cells.

### MiR‐30a is down‐regulated in mouse liver fibrotic tissues induced by BDL or CCL_4_

**Figure 5:**
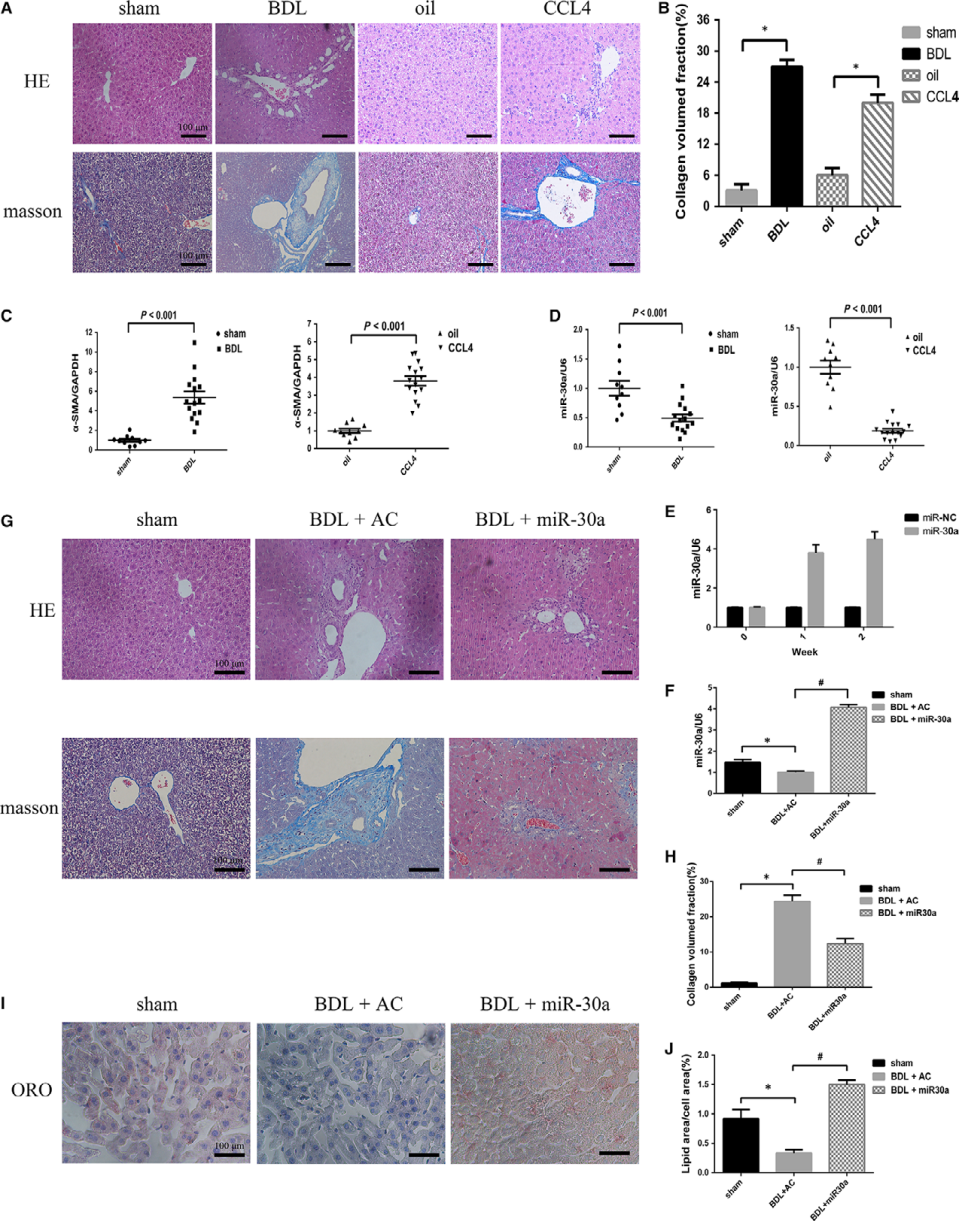
MiR‐30a is down‐regulated *in vivo*, and miR‐30a prevents BDL‐induced hepatic fibrosis in mice. Mouse liver samples dyed with HE or Masson's trichrome (**A**) and semi‐quantitative measurement of Masson's staining (**B**). *P < 0.05 *versus* the control group. RT‐PCR (**C**) revealed an increase in α‐SMA expression in mouse liver fibrotic tissues. TaqMan miRNA assay (**D**) revealed that miR‐30a level was lower in mouse liver fibrotic tissues. *P* < 0.001 *versus* the control group. (**E**) The effectiveness of miR‐30a delivery to the liver of mice and uniform miR‐30a expression was tested at week 1 and week 2. (**F**) MiR‐30a levels were examined. Mouse livers stained with HE or Masson's trichrome (**G**) and semi‐quantitative measurement of Masson's trichrome staining (**H**). (**I** and **J**) Preventive effects of miR‐30a against BDL‐induced hepatic fibrotic tissue changes by ORO staining in mice. (*P < 0.05 *versus* sham; #*P* < 0.05 *versus* BDL + AC.).

Hepatic fibrosis models were developed in mice by induction with BDL (*n* = 15) (sham‐operated as control group, *n* = 10) or CCL_4_ (*n* = 15) (olive oil as control group, *n* = 10). Both BDL and CCL_4_ caused obvious fibrous tissues proliferation of interlobular portal areas, as demonstrated by Masson's trichrome staining from a histological perspective (Fig. 5A). Quantification of collagen content showed that the CVF increased in BDL‐induced fibrotic mice (by 8.76 ± 0.22‐fold *versus* the sham‐operated group, *P *<* *0.05) and CCL_4_‐induced fibrotic mice (by 3.32 ± 0.13‐fold *versus* the olive oil group, *P *<* *0.05) (Fig. 5B). Liver fibrosis is mainly characterized by α‐SMA overexpression 25. Our RT‐PCR data revealed that α‐SMA expression was strongly increased in BDL‐induced liver fibrotic tissues (by 5.36 ± 0.61‐fold *versus* the sham group, *P *<* *0.001) and CCL_4_‐induced liver fibrotic tissues (by 3.79 ± 0.26‐fold *versus* the olive oil group, *P *<* *0.001) (Fig. 5C), whereas miR‐30a expression was down‐regulated in BDL‐induced liver fibrotic tissues (by 0.27 ± 0.03‐fold *versus* the sham‐operated group, *P *<* *0.001) and CCL_4_‐induced liver fibrotic tissues (by 0.19 ± 0.02‐fold *versus* the olive oil group, *P *<* *0.001) (Fig. 5D).

### MiR‐30a prevents BDL‐induced hepatic fibrosis in mice

**Figure 6:**
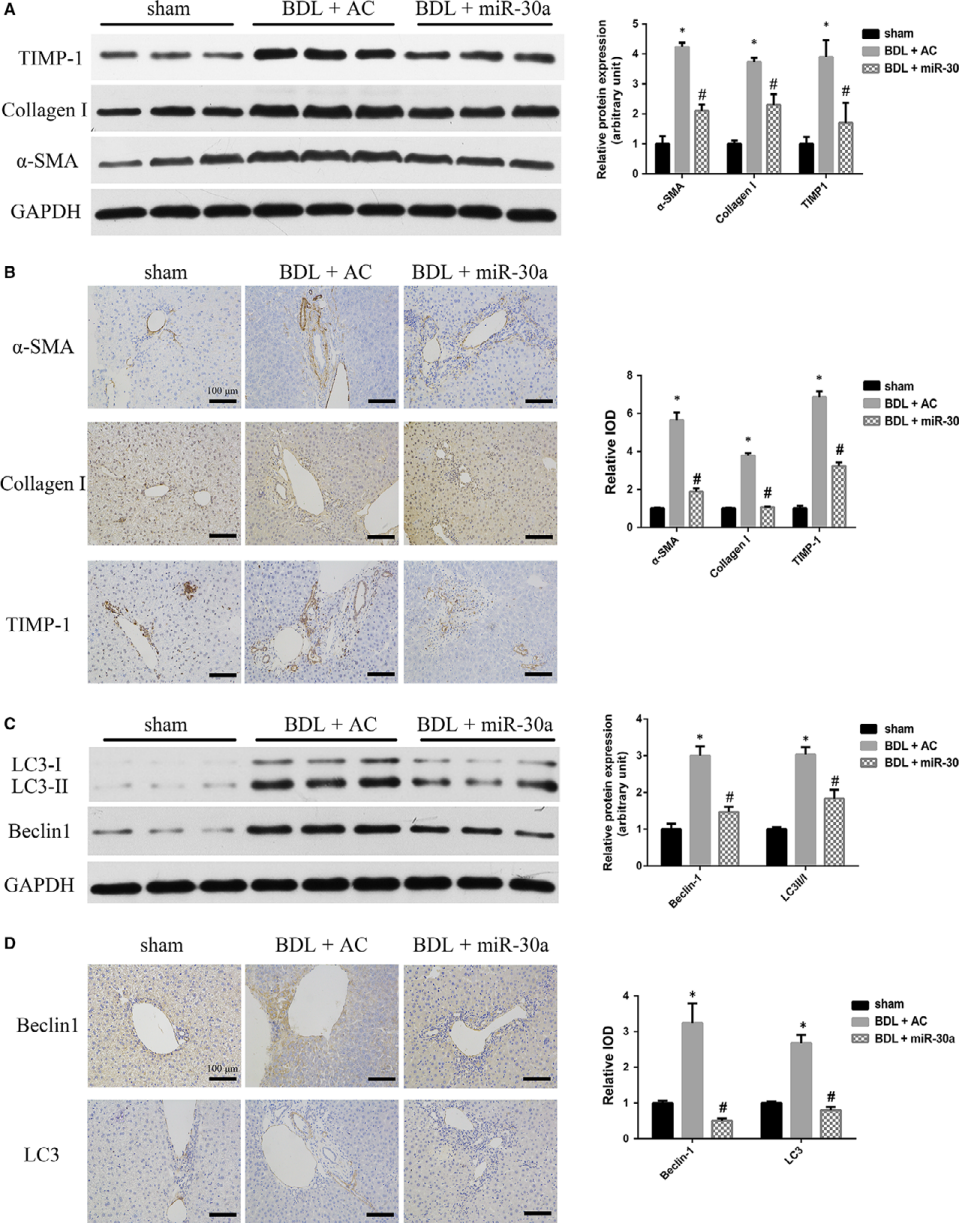
MiR‐30a ameliorates fibrosis in BDL‐induced mouse liver fibrotic tissues. Effects of BDL and transduction with miR‐30a on hepatic protein expression of α‐SMA, TIMP‐1 and Collagen I by (**A**) Western blotting and (**B**) immunohistochemistry. Effects of BDL and miR‐30a overexpression on hepatic protein expression of Beclin1 and LC3 by (**C**) Western blotting and (**D**) immunohistochemistry. *P < 0.05 *versus* sham; #*P* < 0.05 *versus* BDL + AC.

Compared with CCL_4_‐induced group, BDL‐induced group possesses the following advantages: obvious fibrous tissues proliferation and the experiment time to be shorter. Therefore, we selected BDL‐induced group as the experimental model in the further mechanism research. In our study, the ago‐miR‐30a and AC were transfected into C57BL/6 mice, and the efficacy of miR‐30a expression was tested at week 1 and week 2 (Fig. 5E). Overexpression of miR‐30a was proved in the livers of miR‐30a‐transfected mice (Fig. 5F). BDL caused fibrous tissues proliferation of interlobular portal areas and induced the formation of pseudo lobules and tubercles, while excessive expression of miR‐30a alleviated these phenomena (Fig. 5G). As shown in Figure 5H, the CVF was increased in BDL‐induced fibrotic mice compared with that in the sham group, whereas miR‐30a overexpression reduced the secretion of collagen (by 0.49 ± 0.03‐fold *versus* the BDL + AC group, *P *<* *0.05). ORO staining revealed a decrease number of LDs in BDL‐induced mouse liver tissues compared with those in the sham group, whereas aberrant expression of miR‐30a increased the number of LDs (by 4.50 ± 0.22‐fold *versus* the BDL + AC group, *P *<* *0.05) (Fig. 5I and J). To further make sure the anti‐fibrotic effect of miR‐30a in BDL‐treated mice, we measured the serum fibrosis marker hyaluronic acid (HA) and found that it was significantly lower compared to the control group (BDL + AC) (Table 1). Moreover, serum aspartate aminotransferase (AST) and alanine aminotransferase (ALT) were pronouncedly suppressed in BDL‐induced liver fibrosis model by treatment with ago‐miR‐30a (Table 1), compared to the control group. The decrease in AST and ALT levels after miR‐30a treatment indicates a hepatic protective effect of miR‐30a on liver fibrogenesis. Introduction of miR‐30a significantly down‐regulated α‐SMA, TIMP‐1 and Collagen I expression in BDL‐induced mouse liver fibrotic tissues (Fig. 6A), and this was confirmed by immunohistochemistry (Fig. 6B). These findings indicate that miR‐30a prevents liver fibrosis by suppressing proteins involved in fibrogenesis.

**Table 1:** Effect of miR‐30a on serum markers in BDL‐induced liver fibrosis

	HA	AST(U/L)	ALT(U/L)
Sham	11.4 ± 1.2	32.8 ± 1.3	21.7 ± 1.7
BDL + AC	502.5 ± 25.9	731.4 ± 41.7	540.4 ± 35.2
BDL + miR‐30a	386.5 ± 38.2^a^	356.8 ± 26.3^a^	412.6 ± 30.4^a^

Date are mean + S.E.M, *n* = 3.

HA, hyaluronic acid; AST, aspartate aminotransferase; ALT, alanine aminotransferase.

a *versus* BDL + AC.

*P *<* *0.05.

### MiR‐30a suppresses the expression of Beclin1 and LC3‐II/I in BDL‐induced mouse liver fibrotic tissues

The effect of miR‐30a on the expression of the Beclin1 *in vivo* was examined by Western blot and immunohistochemistry. As shown in Fig. 6C and D, Beclin1 was strongly up‐regulated in liver fibrotic tissues in BDL‐treated mice. Additionally, the LC3‐II/I ratio increased after BDL treatment, suggesting that the number of autophagosomes was increased in fibrotic livers. By contrast, BDL‐treated mice supplemented with miR‐30a showed a remarkable down‐regulation of Beclin1 and decreased LC3‐II/I in the fibrotic tissues. These results supported the specific inhibitory effect of miR‐30a on autophagy in liver fibrosis.

## Discussions

Liver fibrosis is a dynamic process of healing and scarring in response to chronic liver injury. As an outcome of long‐lasting damage, liver fibrosis may progress to liver cirrhosis, liver cancer and liver failure 26. Aetiological treatment, fpr example, antiviral treatment for chronic hepatitis B and C, can slow or halt liver fibrosis progression 27. MiRNA‐mediated silencing of target gene expression has been shown to prevent and reverse liver fibrosis 28. The goal of the present study was to analyse the potential anti‐fibrotic effect of miR‐30a.

Our *in vitro* tests demonstrated that miR‐30a was down‐regulated in activated HSCs induced by TGF‐β1 (Fig. 1B), mainly decreased in cytoplasm (Fig. 1C). It has been reported that miR‐30a could be tested in the exosomes from cardiomyocytes 29 and breast cancer cells MCF‐7 30. In addition, our experiments showed that miR‐30a could also be tested in exosomes produced by LX‐2 cells, and miR‐30a expression was similarly down‐regulated in exosomes of TGF‐β1‐treated LX‐2 cells (Fig. 1F). Our *in vivo* tests also showed that miR‐30a was strikingly down‐regulated in mouse liver fibrotic tissues, compared with the normal liver tissues (Fig. 5D). Our findings *in vitro* and *in vivo* suggested that there may be a close link between miR‐30a and liver fibrogenesis. MiR‐30a was reported to act as a negative regulator in myocardial fibrosis 14 and peritoneal fibrosis 15; however, little is known about the contribution of miR‐30a to hepatic fibrosis.

To examine the effect of miR‐30a on hepatic fibrosis *in vitro*, we transduced miR‐30a mimics into HSCs. In this study, we found that overexpression of miR‐30a suppressed the expression of α‐SMA, TIMP‐1, and Collagen I and caused a growth arrest in HSCs (Fig. 2B–D), suggesting that miR‐30a might negatively regulate liver fibrosis by targeting the process of matrix synthesis through inhibiting the activation of HSCs and reducing the number of HSCs *via* suppressing cell proliferation.

To further determine the biological role of miR‐30a in liver fibrosis *in vivo*, a study on mouse liver fibrosis model was applied. We successfully delivered the miR‐30a agomir into BDL‐induced disease livers (Fig. 5F). In the 2‐week BDL‐induced liver fibrosis model, overexpression of miR‐30a notably decreased the severity of hepatic fibrosis, as evidenced by reduced collagen deposition and collagen content (Fig. 5G and H) and the serum fibrosis marker HA (Table 1). In addition, the decrease in AST and ALT levels after miR‐30a treatment indicates a hepatoprotective effect of miR‐30a on liver fibrotic process (Table 1). Consistent with the finding in HSCs, overexpression of miR‐30a inhibited the expression of α‐SMA, TIMP‐1 and Collagen I in livers, compared with the control group (BDL + AC) (Fig. 6A and B), which is more likely the result of reduced activation of HSCs.

We further investigated the possible mechanism by which miR‐30a modulated the activation of HSCs. Autophagy is an evolutionarily conserved self‐digesting process in which cytoplasmic material is sequestered within cytosolic double‐membraned vesicles termed autophagosomes and delivered to the lysosome for degradation 31. A recent study showed that HSCs activation is suppressed by an autophagy inhibitor 32. To the best of our knowledge, miR‐30a is the miRNA that implicated in fibrosis and autophagy. We speculate that miR‐30a may ameliorate hepatic fibrosis by regulating the activity of autophagy. To investigate the influence of miR‐30a on autophagy, Western blot analysis was applied and our results indicated that the levels of LC3‐II/I ratio were decreased in miR‐30a‐treated cells (Fig. 3F). The decrease of autophagic flux in miR‐30a‐treated cells was confirmed by a tandem mRFP‐GFP‐LC3 fluorescence assay (Fig. 3C). Compared with the control group (treated with miR‐NC), the experimental group (treated with miR‐30a mimics) showed a fewer number of autophagic vacuoles by electron microscopy (Fig. 3D). LDs are characteristic of resident HSCs and their presence and induction suppress HSCs activation 33, 34. Autophagy contributes to the intracellular catabolism of lipids in HSCs 8. Our data revealed an increased number of LDs in LX‐2 cells treated with miR‐30a mimics (Fig. 3D and E) and transduction of miR‐30a increased the number of LDs (Fig. 5I and J) compared with the control group (BDL + AC). These data indicated that miR‐30a may prevent HSC activation by suppressing autophagy and increasing LD accumulation in HSCs.

Beclin1, the mammalian homologue of yeast ATG6, was recently reported to be a target of miR‐30a. MiR‐30a up‐regulation inhibits Beclin1‐mediated autophagy 17, whereas miR‐30a down‐regulation activates Beclin1‐mediated autophagy 35. Beclin1 possesses seed‐matching sites with miR‐30a at 3′‐UTR in LX‐2 cells (Fig. 3A and B), and we showed that miR‐30a overexpression significantly decreased Beclin1 level in HSCs (Fig. 3F and G). Moreover, miR‐30a dramatically down‐regulated Beclin1 expression in fibrotic animal models, indicating the translational repression of Beclin1 by miR‐30a (Fig. 6C and D). Knock‐down of Beclin1 in HSCs led to the down‐regulation of α‐SMA, TIMP‐1 and Collagen I and the autophagy markers LC3‐II/I protein expression (Fig. 4B and C). The decrease of autophagic flux in Beclin1 knock‐down HSCs was confirmed by tandem mRFP‐GFP‐LC3 fluorescence assay (Fig. 4D) and electron microscopy (Fig. 4E). ORO staining and electron microscopy revealed an increased number of LDs in LX‐2 cells treated with si‐Beclin1 (Fig. 4E and F). These results suggest a potential mechanism by which miR‐30a inhibits hepatic fibrosis by negatively regulating the Beclin1 signalling pathway *via* interaction with Beclin1. This information highlights the therapeutic potential and underlying mechanism of miR‐30a in inhibiting the Beclin1 pathway to prevent and treat liver fibrosis.

**Figure 7:**
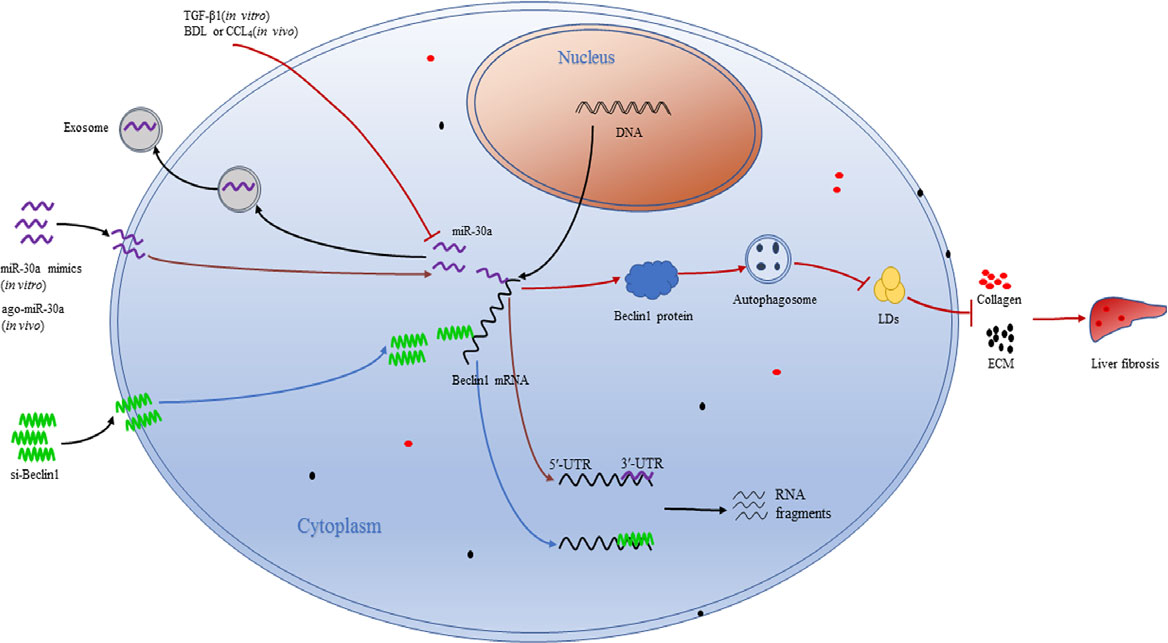
Schematic summary of the anti‐fibrotic effect of miR‐30a in hepatic stellate cells. TGF‐β1 down‐regulates miR‐30a levels in HSCs and LX‐2‐exosomes, BDL or CCL4 down‐regulates miR‐30a levels in HSCs. MiR‐30a or si‐Beclin1 down‐regulates Beclin1 expression (Beclin1 mRNA is degraded into RNA fragments), decreases the number of autophagosomes, promotes lipid droplets (LDs) accumulation, stimulates extracellular matrix (ECM) degradation, suppresses collagen production and results in the amelioration of liver fibrosis.

This is the first study to explore the relationships between miR‐30a, autophagy and hepatic fibrosis. As our work showed that miR‐30a is down‐regulated in TGF‐β1 activated HSCs and LX‐2‐exosomes, and mouse hepatic fibrosis tissues. MiR‐30a acts as an anti‐fibrotic factor and has therapeutic potential in hepatic fibrosis by repressing the activation of HSCs, resulting in stimulating matrix degradation and suppressing collagen production. We demonstrated that the molecular basis of the anti‐fibrotic effect of miR‐30a is to directly inhibit Beclin1, leading to the inactivation of the Beclin1 signalling pathway and ultimately the inhibition of autophagy in HSCs (Fig. 7). MiR‐30a inhibited autophagy in both HSCs and BDL‐treated mice. Knock‐down of Beclin1 inhibited autophagy and fibrogenesis in LX‐2 cells. These findings indicate that overexpression of miR‐30a may prevent liver fibrogenesis by an autophagy‐dependent pathway, thus representing a new and promising therapeutic target for liver fibrosis.

## Conflict of interest

All authors declare no potential conflicts of interest with respect to the research, authorship and/or publication of this article.

## Supporting information


**Table S1** The consequences of miR‐30a and si‐Beclin1.

**Table S2** RT‐PCR primers used in this study.
