# Supplementary material for: MicroRNA‐30a ameliorates hepatic fibrosis by inhibiting Beclin1‐mediated autophagy
Source: J Cell Mol Med. 2017 Aug 1;21(12):3679–92. doi: 10.1111/jcmm.13278 (PMC5706581; doi:10.1111/jcmm.13278)
Supplement: Supplementary file 1 — Table S1 The consequences of miR‐30a and si‐Beclin1. [file JCMM-21-3679-s001.docx]

| miR-30a mimics | |
| --- | --- |
| sense | UGUAAACAUCCUCGACUGGAAG |
| antisense | UCCAGUCGAGGAUGUUUACAUU |
| miR-NC | |
| sense | UUCUCCGAACGUGUCACGUTT |
| antisense | ACGUGACACGUUCGGAGAATT |
| miR-30a agomir | |
| sense | UGUAAACAUCCUCGACUGGAAG |
| antisense | UCCAGUCGAGGAUGUUUACAUU |
| AC | |
| sense | UUCUCCGAACGUGUCACGUTT |
| antisense | ACGUGACACGUUCGGAGAATT |
| si-Beclin1 | |
| sense | CUGGACACGAGUUUCAAGATT |
| antisense | UCUUGAAACUCGUGUCCAGTT |
| si-NC | |
| sense | UUCUCCGAACGUGUCACGUTT |
| antisense | ACGUGACACGUUCGGAGAATT |

Abbreviation: miR-NC, miRNA mimic negative control; AC, agomir control.
