# Supplementary material for: MicroRNA‐30a ameliorates hepatic fibrosis by inhibiting Beclin1‐mediated autophagy
Source: J Cell Mol Med. 2017 Aug 1;21(12):3679–92. doi: 10.1111/jcmm.13278 (PMC5706581; doi:10.1111/jcmm.13278)
Supplement: Supplementary file 2 — Table S2 RT‐PCR primers used in this study. [file JCMM-21-3679-s002.docx]

| Gene symbol | Forward | Reverse |
| --- | --- | --- |
|  |  |  |
| Human mRNA |  |  |
| α-SMA | CGTGGCTATTCCTTCGTTAC | TGCCAGCAGACTCCATCC |
| Collagen I | GAGGGCCAAGACGAAGACATC | CAGATCACGTCATCGCACAAC |
| TIMP-1 | CTTCTGCAATTCCGACCTCGT | ACGCTGGTATAAGGTGGTCTG |
| Beclin1 | CCATGCAGGTGAGCTTCGT | GAATCTGCGAGAGACACCATC |
| LC3 | GATGTCCGACTTATTCGAGAGC | TTGAGCTGTAAGCGCCTTCTA |
| GAPDH | GGAGCGAGATCCCTCCAAAAT | GGCTGTTGTCATACTTCTCATGG |
|  |  |  |
| Rat mRNA |  |  |
| α-SMA | GCTCTGTAAGGCGGGCTTTG | ACGAAGGAATAGCCACGCTCA |
| Collagen I | GGAGAGAGCATGACCGATGG | GGGACTTCTTGAGGTTGCCA |
| TIMP-1 | TGCTCAAAGGATTCGACGCT | AGCAGGGCTCAGATTATGCC |
| Beclin1 | GAATGGAGGGGTCTAAGGCG | CTTCCTCCTGGCTCTCTCCT |
| LC3 | GAAGACCTTCAAACAGCGCC | CTTGGTCTTGTCCAGGACGG |
| GAPDH | ACAGCAACAGGGTGGTGGAC | TTTGAGGGTGCAGCGAACTT |
|  |  |  |
| Mouse mRNA |  |  |
| α-SMA | GGCTCTGGGCTCTGTAAGG | CTCTTGCTCTGGGCTTCATC |
| Collagen I | GCTCCTCTTAGGGGCCACT | CCACGTCTCACCATTGGGG |
| TIMP-1 | GCAACTCGGACCTGGTCATAA | CGGCCCGTGATGAGAAACT |
| Beclin1 | ATGGAGGGGTCTAAGGCGTC | TCCTCTCCTGAGTTAGCCTCT |
| LC3 | TTATAGAGCGATACAAGGGGGAG | CGCCGTCTGATTATCTTGATGAG |
| GAPDH | AGGTCGGTGTGAACGGATTTG | GGGGTCGTTGATGGCAACA |

**RT-PCR primers used in this study.**
